# Supplementary material for: Whole-Genome Analysis Reveals the Dynamic Evolution of Holocentric Chromosomes in Satyrine Butterflies
Source: Genes (Basel). 2023 Feb 8;14(2):437. doi: 10.3390/genes14020437 (PMC9956908; doi:10.3390/genes14020437)
Supplement: Supplementary file 1 [file genes-14-00437-s001.zip › genes-2142369-supplementary.pdf]

Supplementary file
Table S1. Studied species and chromosomes.

Maniola jurtina
median GC%: 36.83

Chromosome	GenBank #	Size (Mb)	GC%
1	HG995207.1	17.320	36.8
2	HG995209.1	17.190	36.6
3	HG995210.1	16.760	36.8
4	HG995211.1	16.670	36.8
5	HG995212.1	16.230	36.7
6	HG995213.1	16.200	36.8
7	HG995214.1	15.710	36.3
8	HG995215.1	15.420	36.9
9	HG995216.1	15.230	37.0
10	HG995217.1	15.170	36.6
11	HG995218.1	15.110	36.8
12	HG995219.1	15.090	36.7
13	HG995220.1	14.830	36.9
14	HG995221.1	14.700	36.9
15	HG995222.1	14.150	36.9
16	HG995223.1	14.030	36.8
17	HG995224.1	13.720	36.9
18	HG995225.1	13.540	36.9
19	HG995226.1	13.290	37.1
20	HG995227.1	13.280	37.0
21	HG995228.1	12.260	37.3
22	HG995229.1	12.130	36.9
23	HG995230.1	10.030	37.2
24	HG995231.1	9.430	37.4
25	HG995232.1	8.570	37.3
26	HG995233.1	7.750	38.7
27	HG995235.1	6.950	37.7
28	HG995236.1	6.660	38.1
Z	HG995208.1	17.210	36.9
W	HG995234.1	7.350	38.3
unplaced		0.04	37.4

Erebia ligea
median GC%: 37.3942

Chromosome	GenBank #	Size (Mb)	GC%
1	OU785219.1	22.720	37.2
2	OU785220.1	22.110	37.3
3	OU785221.1	22.010	37.4
4	OU785223.1	21.420	37.3
5	OU785224.1	20.970	37.3

6	OU785225.1	20.770	37.1
7	OU785226.1	20.520	37.100
8	OU785227.1	20.110	37.400
9	OU785228.1	20.060	37.300
10	OU785229.1	19.310	37.400
11	OU785230.1	19.220	37.300
12	OU785231.1	19.150	37.200
13	OU785232.1	18.890	37.3
14	OU785233.1	18.500	37.4
15	OU785234.1	18.360	37.3
16	OU785235.1	17.540	37.4
17	OU785236.1	17.220	37.7
18	OU785237.1	16.820	37.2
19	OU785238.1	16.820	37.4
20	OU785239.1	16.640	37.5
21	OU785240.1	15.510	37.3
22	OU785241.1	15.260	37.6
23	OU785242.1	12.470	37.7
24	OU785243.1	12.370	38.2
25	OU785244.1	11.940	37.5
26	OU785245.1	10.280	37.7
27	OU785246.1	9.220	37.8
28	OU785247.1	8.140	38.1
Z	OU785222.1	21.640	37.3
unplaced		0.390	41.4

Erebia aethiops
median GC%: 37.2211

Chromosome	GenBank #	Size (Mb)	GC%
1	OV281080.1	33.250	37.1
2	OV281081.1	32.760	37.1
3	OV281082.1	32.720	37.2
4	OV281083.1	30.440	37.1
5	OV281084.1	30.010	37.5
6	OV281085.1	26.260	37.4
7	OV281086.1	25.860	37.4
8	OV281087.1	23.960	37.2
9	OV281088.1	20.720	37.3
10	OV281089.1	20.450	37.1
11	OV281090.1	20.105	37.3
12	OV281091.1	19.450	37.3
13	OV281092.1	19.300	37.3
14	OV281093.1	18.420	37.2
15	OV281094.1	17.950	37.3
16	OV281095.1	17.050	37.3
17	OV281096.1	15.920	37.4

18	OV281097.1	15.760	37.7
Z	OV281079.1	37.950	36.8
W	OW818193.1	3.110	37.7
unplaced		11.97	37.5
